# Medical and Philosophical News

**Published:** 1783

**Authors:** 


### SECTION III.

### Medical and Philosophical News.

AT a public meeting of the Royal Medical Society at Paris, on the 26th of Auguſt 1783, the eulogium of the late Sir John Pringle, bart. written by M. Vicq d'Azyr was read; as was likewiſe a memoir by Meſſrs. de Laſſone and Cornette, concerning a new method of preparing paring at a ſmall expence, and in a ſhort time, an extract of opium by digeſtion.

The prize relative to the ſcurvy, which we formerly announced (*vol*. 3, *p*. 419), was divided between M. Goguelin, phyſician at Moncontour in Brittany, and M. Bougourd, phyſician at St. Malo, authors of two diſſertations, which in the opinion of the ſociety were equally entitled to the premium.—Several diſſertations have been received by the ſociety on another of the prize queſtions formerly propoſed, viz. The chemical analyſis of antiſcorbutic remedies procured from the tribe of cruciform plants (*See* vol. 3. *p*.198); but as none of theſe pieces are ſufficiently ſatisfactory, the ſociety have thought it right to announce the ſame queſtion a ſecond time. Diſſertations on this ſubject will be received by the ſecretary till the 1ſt of January 1785.

The Society likewiſe offer a premium of 600 livres to the writer who ſhall "aſcertain the ad-"vantages and diſadvantages of the Peruvian "bark adminiſtered in the treatment of the dif-"ferent ſpecies of remittent fevers." The diſſertations on this ſubject muſt be ſent to M. Vicq d'Azyr, ſecretary of the Society, before the 1ſt of May 1785.

At

---

At a meeting of the Royal Academy of Sciences at Paris, on the 12th of November 1783, M. Lavoiſier read a very curious eſſay on the analyſis of water. The experiments of which he gave an account ſeem to prove, that water is not a ſimple, elementary ſubſtance, as hath hitherto been imagined, but a compound body, ſuſceptible of decompoſition and recompoſition. His opinions on this ſubject originated, it ſeems, from ſome inquiries in which he engaged with the late M. Bucquet in 1777. He was then ſurprized at obſerving that the combuſtion of inflammable air obtained by diſſolving iron in vitriolic acid, in cloſe veſſels, afforded no mark of fixed air, or acid.—Mr. Cavendiſh had before remarked, that by burning inflammable air in a cloſe veſſel, the ſides of the veſſel were covered with a ſenſible moiſture.—M. Lavoiſier and M. de la Place were deſirous of making farther experiments on this ſubject. They began with mixing thirty pints of inflammable air and fifteen of dephlogiſticated air under a glaſs bell plunged in quickſilver. It was not long before the inner ſurface of the bell began to be obſcured, and ſoon after drops of water were ſeen feen trickling down its fides to the furface of the mercury. The water procured by this procefs was as pure as diftilled water, and nearly equal in weight to the two airs united. It feemed to follow from this experiment, that water obtained in this way might be confidered as a compound fubftance, the conftituent parts of which are the two fpecies of air above-mentioned. To confirm this opinion there remained only to be able to decompofe water. This is what M. Lavoifier profeffes actually to have done, by mixing iron filings and water. In this experiment, which he deems conclufive, the inflammable air of the water was extricated, and its other conftituent principle, viz. its dephlogifticated air, uniting with the filings of iron converted them into a calx. From thefe experiments he is led to affert, that air and water are one and the fame element.—M. Lavoifier applies this difcovery to the explanation of many phænomena in the decompofition of bodies in general, the folution and calcination of metals, fermentation, vegetation, &c. He imagines that in fermentation water furnifhes the portion of inflammable air which conftitutes the vinous part; and in vegetation, that which renders vegetables combuftible.

---

Mr.

Mr. Scheele has lately communicated to profeſſor Crell, of Helmſtadt, an account of ſome experiments which prove that the colouring matter of the blood is compoſed of volatile alkali, phlogiſton, and fixed air.

---

Extract of a letter from Dr. William Halliday, to Dr. William Saunders, phyſician in London, dated Tubingen, Nov. 30, 1783.

" Since the publication of your obſervations

" on the red bark many of the phyſicians here

" have been induced to make trial of its ſupe-

" rior efficacy, but their expectations have been

" too often baffled by the avarice of the drug-

" giſts. It is true they have a ſort of bark here

" which they call *red* bark, and which is dif-

" ferent from that hitherto uſed, but in the

" different ſhops I have ſeen there is nothing

" like that which you uſed to ſhew and deſcribe

" to your pupils. Indeed, I believe, that little

" or none of the real kind has yet appeared in

" this country, at leaſt in Wirtemberg. In the

" capital of Wirtemberg I had occaſion a few

" weeks ago to make uſe of the Peruvian bark,

" and

" and to be the more certain of obtaining a ge-
" nuine fort, I went to different apothecaries,
" but inftead of meeting with what I wanted,
" I was fhewn a kind of bark, that was indeed
" externally red, but upon breaking it, it was
" found to be of a grey colour internally, and
" of a woody texture, and moft of the pieces
" were rotten. This fophiftication is probably
" practifed by the Dutch, as moft of the bark
" employed here is procured from Holland.—
" Three days ago I was requefted by the in-
" fpecting phyfician of this place to look at
" fome medicines lately arrived, amongft which
" was a yellowifh coloured bark, a little groffer
" than the ordinary bark, and which was faid
" to be the genuine red bark. All prefent
" were furprized when I faid it was not at all
" like that defcribed by you; and to convince
" them of this, I fhewed them fome fpecimens
" of red bark which I had brought with me
" from England."——In addition to what is here faid, concerning the fophiftication of the red bark, it may not be improper to add, that the adulteration of this valuable drug is not confined to the continent. There are great quantities of a fpurious fort, particularly in powder, now on ſale in this country, ſo that practitioners cannot be too careful in their choice of this article.

---

The letter of which we have given an extract in the preceding article was accompanied with an Engliſh MS. tranſlation by Dr. Halliday, of a paper containing obſervations and experiments on the red bark by Dr. Cothenius, firſt phyſician to the king of Pruſſia, read at a meeting of the academy of ſciences at Berlin on the 4th of July 1783. In this eſſay, which has been obligingly put into our hands by Dr. Saunders, the author obſerves, that Dr. Gleditch, profeſſor of botany at Berlin, has now in his poſſeſſion, a ſpecimen of red bark given to him in 1733, in a paper on which was written "*Cortex* "*Peruvianus Certus*, approuvé par Meſſieurs "Tournefort, Balduin, and Barbie," and Dr. Cothenius himſelf remembers that ſixty years ago there was no other than the red bark to be found in the apothecaries ſhops in Pomerania, and that at that time three or four doſes of it generally uſed to cure an intermittent. In the year 1758, the late earl Mariſchal returning from Spain brought with him, as a preſent to the the author, a pound of red bark, which he aſſured him was preferable to all the other ſorts, and the only kind uſed by the Spaniſh nobility. —Dr. Cothenius has obſerved, that the red is ſpecifically heavier than the common bark. A glaſs full of the former, in powder, weighed ʒv, and of the latter ʒiv, gr. xlv. A chemical analyſis of eight ounces of red bark yielded,

| | | |
|---|---|---|
| of a vegetable alcaline ſalt | | 22 grains, |
| — vitriolated tartar | — | 6 |
| — earth of iron | — — | 8 |
| — calcareous earth | — | 48 |
| — ſelenites | — — — | 6 |

From the ſame quantity of common bark were obtained,

| | | |
|---|---|---|
| of a vegetable alcaline ſalt | | 30 grains, |
| — vitriolated tartar | — | 4 |
| — earth of iron | — — | 4 |
| — calcareous earth | — | 37 |
| — ſelenites | — — — | 6 |

---

In a former volume of our Journal (vol. III. p. 222.) ſome account was given of the efficacy of opium in the cure of the lues venerea. It ſeems that in caſes of this ſort this remedy has for ſome time paſt been in general uſe in the

 milimilitary hoſpitals in North America, and in the greater number of caſes was neither accompanied nor preceded by the uſe of mercury. Dr. Saunders, who is at preſent engaged in trying the effects of this new mode of treatment in different patients, in Guy's Hoſpital, and who has promiſed to inform us of the reſult of his obſervations on this ſubject, has already cured one patient, by this method, who had ſeveral deep, ill-conditioned chancres. The ſores were waſhed with a ſolution of opium in water, and the patient began with ſwallowing three grains of opium in the courſe of a day. The doſes were gradually increaſed till he took twelve grains in that ſpace of time, and in ten days all the venereal ſymptoms were removed. Dr. Saunders is not yet able to aſcertain how far opium may be relied on in caſes of this ſort independently of the uſe of mercury, but thinks it may be employed with great advantage, not only as a means of taking off irritation, and of courſe promoting the healing of venereal ſores, which is always a deſirable object, but likewiſe with a view to check the ſalivating power of mercury, and determine it to the ſkin.

---

The plant which produces Aſſafœtida flowered laſt ſummer in the botanic garden at Edinburgh, and bore ſeveral ſeeds. A deſcription and engraving of it are ſoon to be publiſhed.

---

Extract of a letter from Dr. Robert Willan, phyſician in London, to Dr. Simmons.

" In the Medical Journal, (vol. III. p. 430) was inſerted the extract of a letter from Dr. Veling, relating to a new ſpecies of Byſſus found in the ſulphur waters of Aix la Chapelle and Burdſcheid, which he ſuppoſed might be the ſame with that deſcribed in my Obſervations on the ſulphur water at Croft, in Yorkſhire. The doctor, according to his promiſe, has done me the favour to ſend a ſpecimen of it, which I examined, with the aſſiſtance of Mr. Dickſon, thro' Sir Joſeph Banks's beſt magnifier. We were ſurprized to find it a new ſpecies, totally different from the Croft Byſſus, not being compoſed, like that, of paraſitical filaments growing in a plumoſe form on the threads of conferva rivularis, but conſiſting of long, regular filaments very much interwoven and coiled together.

The

The following botanical character, therefore, seems proper for this plant;

*Byssus filamentis simplicissimis, æqualibus, albis.* The Byssus of the waters of Croft, Dinsdale, &c. was thus arranged and described with the approbation of the late Dr. Solander—*Byssus lanuginosa filamentis simplicibus, tenuissimis, albis; habitat in fontibus præcipue sulphureis, confervis adhærens, lapidesque &c. obtegens.*"

---

The Royal Society have this year distributed two Copleian medals. Of these one was presented to Mr. Goodricke, for having determined the period of the revolution of the star Algol, a discovery that will claim a distinguished place in the history of astronomy. This young gentleman, who is grandson and heir apparent of Sir John Goodricke, Bart. has been deaf from his infancy, and is one of those pupils who do so much honour to Mr. Braidwood's academy for the deaf and dumb. The other medal was conferred on Mr. Hutchins, governor of Fort Albany in Hudson's Bay, for a discovery of great importance in another branch of science, of which we have already given an account (*page* 205).—The learned president of the society, in a discourse delivered on this occasion, exhibited a very interesting view of the utility and importance of these discoveries.

*New works about to be published.*——I. Two introductory lectures by the late Dr. William Hunter.—II. Experiments and observations to demonstrate the chemical history of the tepid springs of Buxton, by Dr. George Pearson, physician in London.—III. An English translation of M. Fourcroy's *Leçons Elementaires d'Histoire Naturelle et de Chemie* (see p. 332).—IV. At Leipsic, Dr. Franz, professor of physic, is engaged in preparing for the press a work entitled, *Archæologiæ Artis Obstetriciæ et Puerperii*, and likewise new editions, with notes, of the following; viz. 1. Two books of Veterinary physic (*De Medicina Veterinaria*) in Greek, with the Latin translation of Ruellius; 2. The *Ars Veterinaria sive Mulo-medicina* of Flavius Vegetius compared with a MS. at Gotha; 3. Q. Serenus Scammonius *de Re Medica* compared with MSS. at Paderborn, Breslaw, and Leipsic; 4. Æmilius Macer *de herbarum virtutibus* compared with a MS. at Leipsic.—V. Professor Crell, of Helmstadt, proposes to publish a work in German, entitled *Beytrage Zum Chemischen Archive*, which is to contain an account, in chronological order, of all the chemical facts and observations dispersed in the transactions of different academies. Two volumes of this work are to be published annually annually at Leipſic till the whole is completed. He intends to begin with the Philoſophical Tranſactions, the *Acta Eruditorum*, and the memoirs of the Academy of Sciences at Paris. — VI. M. de Quengſy, author of a collection of obſervations on the eye (*ſee p.* 219) intends ſoon to publiſh a work in one volume 8vo. entitled, *Cours d'Operations ſur la Chirurgie des Yeux*, with engravings of inſtruments and practical remarks.

